# Association Between Fracture Morphology and Preoperative Acute Kidney Injury in Patients with Intertrochanteric Fracture

**DOI:** 10.3390/jcm14144999

**Published:** 2025-07-15

**Authors:** Myeong Gu Lee, Kee Hyung Rhyu, Young Soo Chun

**Affiliations:** 1 Department of Orthopaedic Surgery, Kyung Hee University Hospital at Gangdong, Seoul 05278, Republic of Korea; myeonggu.lee@khu.ac.kr; 2 Department of Medicine, Graduate School, Kyung Hee University, Seoul 02447, Republic of Korea; 3 Department of Orthopaedic Surgery, Kyung Hee University Hospital, Seoul 02447, Republic of Korea; khrhyu@empal.com

**Keywords:** hip fractures, acute kidney injury, mortality

## Abstract

**Background**: While postoperative acute kidney injury (AKI) in patients with hip fracture has been investigated, the relationship between fracture morphology and the incidence of preoperative AKI remains unclear. This study aimed to investigate the association between fracture morphology and the incidence of preoperative AKI, as well as its impact on in-hospital mortality and length of hospital stay. **Methods**: A retrospective analysis was conducted on 462 patients with intertrochanteric fractures treated at a single university hospital between January 2018 and December 2023. The fractures were categorized based on radiographic morphology into two groups: simple fractures and comminuted fractures. Preoperative AKI was diagnosed using KDIGO criteria based on serum creatinine levels measured at the time of emergency department admission. Demographic characteristics and comorbidities were collected. Clinical outcomes included time to surgery, length of hospital stay, and in-hospital mortality. Multivariable logistic regression was used to identify independent risk factors for preoperative AKI. **Results**: Among 462 patients, 66 (14.3%) developed preoperative AKI. The incidence of AKI was significantly higher in the comminuted fracture group than in the simple fracture group (17.5% vs. 10.2%, *p* = 0.037). Multivariable analysis identified comminuted fracture morphology as an independent risk factor for preoperative AKI (OR 2.44, 95% CI 1.19–5.00, *p* = 0.015). Preoperative AKI was also significantly associated with increased in-hospital mortality (OR 4.56, CI 1.40–14.81, *p* = 0.018). **Conclusions**: Comminuted intertrochanteric fracture is significantly associated with an increased risk of preoperative AKI. Preoperative AKI is linked to worse clinical outcomes, including higher in-hospital mortality. These findings emphasize the importance of close monitoring of renal function and proper management of AKI in comminuted fracture group.

## 1. Introduction

Hip fractures in older adults represent a major public health concern, contributing to substantial morbidity, loss of independence, and increased mortality. As the global population ages, the incidence of hip fractures is expected to rise, posing a significant burden on healthcare systems. Among the serious complications observed in this population, acute kidney injury (AKI) is a particularly important concern. The development of AKI in older patients often results from a combination of age-related vulnerability, dehydration, systemic inflammation, and exposure to nephrotoxic agents during hospitalization [1].

Previous studies have reported that the incidence of postoperative AKI in patients undergoing hip fracture surgery ranges from 4.4 to 28.4% [2,3,4,5,6,7,8,9,10,11,12,13,14,15]. Patients who develop AKI are more likely to experience prolonged hospital stays, increased healthcare costs, and higher mortality rates [6,8]. Moreover, the presence of AKI is associated with a three-fold increase in 30-day mortality, with excess mortality persisting up to one-year postoperatively [2].

Despite the growing body of literature on postoperative AKI in patients with hip fracture, limited attention has been given to the incidence and clinical implications of AKI that develops preoperatively. Additionally, most prior studies have focused on risk factors for AKI, such as patient related factors—including advanced age, chronic kidney disease, and other comorbidities—and intraoperative factors [12], while the impact of initial injury-related physiological stressors remains understudied. Although the apparent severity of injury may seem relatively uniform due to the low-energy mechanism of most hip fractures in older patients, underlying fracture characteristics—such as type, stability, and morphology—can differ substantially, influencing initial hidden blood loss and renal perfusion [16,17,18,19].

Intertrochanteric fractures, a common subtype of hip fractures in the elderly, display substantial morphological heterogeneity. These fractures may range from simple two-part configurations to complex, unstable patterns with extensive comminution or reverse obliquity. Intertrochanteric fractures can cause greater hidden blood loss compared to femoral neck fractures [18]. Additionally, unstable intertrochanteric fractures are associated with greater perioperative blood loss and a higher likelihood of requiring transfusion compared to stable intertrochanteric fractures [19]. The degree of fracture comminution has been shown to correlate with the extent of soft tissue damage and bleeding, which can lead to hypovolemia, a key risk factor for prerenal AKI.

The purpose of this study was to investigate the association between fracture morphology and the incidence of preoperative AKI in patients with intertrochanteric fractures. We hypothesized that patients with comminuted fracture morphology would be associated with an increased risk of preoperative AKI, potentially due to increased hemorrhage and hypovolemia. We also aimed to examine whether preoperative AKI influences clinical outcomes, including length of hospital stay and in-hospital mortality.

## 2. Materials and Methods

### 2.1. Study Design and Patients Selection

This retrospective study was approved by the Institutional Review Board of Kyung Hee University Hospital at Gangdong (IRB File No. 2025-03-023; approval date: 15 April 2025) and conducted in accordance with the Declaration of Helsinki. We included all patients who presented to the single university hospital with intertrochanteric fractures between January 2018 and December 2023. All cases were identified using the corresponding ICD-10 diagnostic codes recorded at the time of hospital presentation. Demographic data (age, sex) and clinical history (comorbidities, history of antithrombotic agents use, ambulatory status) were extracted from electronic medical records.

A total of 576 patients were initially identified. Exclusion criteria included chronic kidney disease (CKD), revision surgery, non-acute fractures (defined as injury sustained more than three weeks prior to admission), polytrauma or high-energy trauma in patients under 65 years of age, and pathologic fractures due to metastatic disease. CKD—a well-established risk factor for AKI—was excluded because baseline serum creatinine levels are often difficult to estimate in these patients, making it challenging to accurately diagnose superimposed AKI. Additionally, this study aimed to evaluate the incidence and associated factors of AKI in patients without pre-existing renal disease. To ensure a homogeneous geriatric population, we excluded younger patients with high-energy trauma to minimize the confounding effects of general trauma severity. After applying these criteria, 114 patients were excluded: 68 with CKD, 7 with revision surgery, 4 with non-acute fractures, 32 with high-energy trauma or polytrauma, and 3 with pathologic fractures, resulting in 462 patients eligible for analysis.

### 2.2. Fracture Classification

**Figure 1:**
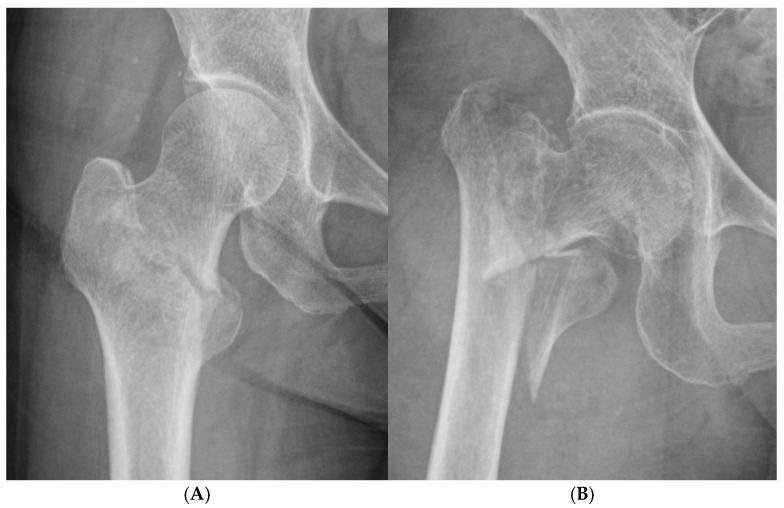
Radiographic classification of intertrochanteric fractures. (**A**) Simple two-part fracture with intact medial cortex. (**B**) Comminuted fracture with disruption of the posteromedial cortex.

Intertrochanteric fracture morphology was categorized into two groups based on the initial radiographs obtained in the emergency department: simple fractures and comminuted fractures. The simple fractures included two-part fractures with intact posteromedial cortex, simple reverse obliquity fractures, and incomplete fractures that did not extend to the medial cortex. Fractures involving comminution, including disruption of the posteromedial cortex, were classified as comminuted fractures (Figure 1). The classification was performed by a single orthopedic surgeon.

### 2.3. Diagnosis of AKI

Preoperative AKI was defined according to the Kidney Disease: Improving Global Outcomes (KDIGO) criteria [20], which are internationally accepted for standardized AKI diagnosis. Serum creatinine values measured upon presentation to the emergency department were used for initial assessment. AKI was diagnosed if there was an increase in serum creatinine of ≥0.3 mg/dl within 48 h or a ≥1.5-fold increase from baseline. For patients without available prior laboratory data, baseline serum creatinine was estimated by assuming an eGFR of 75 mL/min/1.73 m^2^ and applying the MDRD (Modification of Diet in Renal Disease) equation, as recommended by the KDIGO guidelines. Clinical outcomes included time to surgery, length of hospital stay, and in-hospital mortality rate.

### 2.4. Fracture Management and Surgical Procedure

Of the 462 patients included in this study, 447 patients underwent surgery, while 15 patients did not. Reasons for non-operative management included severe medical comorbidities, pre-injury bed-ridden status, and refusal of surgery by the patient or family. Since the focus of our study was on preoperative renal function at the time of hospital admission, all patients were included regardless of surgical treatment.

Surgical procedures were performed on a fracture table under fluoroscopic guidance. Closed reduction was initially attempted with traction and internal rotation. If satisfactory alignment could not be achieved, percutaneous reduction was performed. All surgeries were performed using cephalomedullary nailing. Each patient received either the Proximal Femoral Nail Antirotation (PFNA; DePuy Synthes, West Chester, PA, USA) or the Gamma3 Nail (Stryker, Kalamazoo, MI, USA).

### 2.5. Statistical Analysis

Demographic data, clinical outcomes, and the incidence of preoperative AKI were compared between the two groups using chi-square test for categorical variables, and independent t-test for continuous variables. The normality test of continuous variables was assessed using the Shapiro–Wilk test. To identify factors associated with preoperative AKI, univariable logistic regression analysis was performed for each independent variable, including patient demographics, comorbidities, and intertrochanteric fracture morphology. Variables with a *p*-value < 0.1 in the univariable analysis were subsequently included in a multivariable logistic regression model to adjust for potential confounding factors. The results were reported as odds ratios (ORs) with 95% confidence intervals (CIs). To evaluate the association between preoperative AKI and in-hospital mortality, Fisher’s exact test was used due to the small number of mortality events. Statistical significance for all analyses was defined as a *p*-value < 0.05. All statistical analyses were performed using SPSS statistical program for Windows, version 22.0 (SPSS Inc., Chicago, IL, USA).

## 3. Results

### 3.1. Comparison Between Simple and Comminuted Fracture Groups

**Figure 2:**
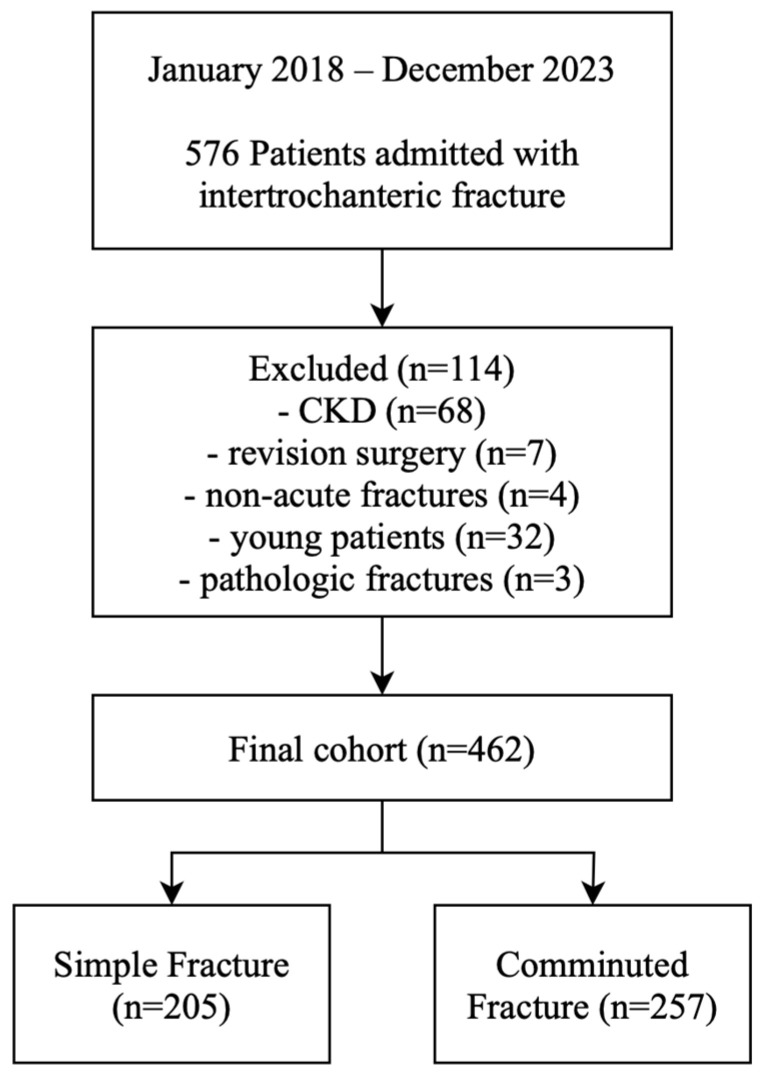
Flowchart of study design.

Among the 462 patients included in this study, 205 were categorized into the simple fracture group and 257 into the comminuted fracture group (Figure 2). In total, 66 (14.3%) developed preoperative AKI. No significant differences were observed between the simple and comminuted fracture groups in terms of sex, age, comorbidities, time to surgery, length of hospital stay, and in-hospital mortality. However, the incidence of preoperative AKI was significantly higher in the comminuted fracture group compared to the simple fracture group (17.5% vs. 10.2%, *p* = 0.037). The mean preoperative hemoglobin level was significantly lower in the comminuted fracture group than in the simple fracture group (10.87 g/dL vs. 11.48 g/dL, *p* < 0.001) (Table 1).

### 3.2. Univariable Analysis of Risk Factors for Preoperative AKI

In univariable logistic regression analysis, the following variables showed a potential association with an increased risk of preoperative AKI (*p* < 0.1): age (OR 1.04; 95% CI, 1.00–1.09; *p* = 0.077), comminuted fracture morphology (OR 2.62; 95% CI, 1.32–5.23; *p* = 0.006), history of cerebrovascular disease (OR 2.16; 95% CI, 0.68–6.79; *p* = 0.095), use of antithrombotic agents (OR 1.88; 95% CI, 0.60–5.93; *p* = 0.069), and use of an ambulation aid (OR 2.41; 95% CI, 1.14–5.08; *p* = 0.021). These variables were included in the multivariable analysis. Other variables, including male sex, hypertension, diabetes mellitus, history of myocardial infarction, dementia, and dyslipidemia, were not significantly associated with preoperative AKI (Table 2).

### 3.3. Multivariable Analysis of Risk Factors for Preoperative AKI

In the final multivariable logistic regression analysis, comminuted fracture morphology remained a significant independent risk factor for preoperative AKI (OR 2.44, 95% CI 1.19–5.00, *p* = 0.015). The use of antithrombotic agents demonstrated a borderline association (OR 1.80, 95% CI 0.89–2.51, *p* = 0.099), while age, history of cerebrovascular disease, and use of ambulation aid were not significant in the final model (Table 3).

### 3.4. Comparison of Clinical Outcomes Between Patients with and Without Preoperative AKI

Due to the small number of in-hospital mortality events, logistic regression analysis was not feasible. Instead, Fisher’s exact test was used to evaluate the association between preoperative AKI and in-hospital mortality. Patients with preoperative AKI had a significantly longer hospital stay compared to those without AKI (20.9 ± 17.0 vs. 15.0 ± 11.5 days, *p* = 0.001). In-hospital mortality occurred in 5 of 66 patients with preoperative AKI (7.6%) and in 7 of 396 patients without AKI (1.8%), demonstrating a significant association between preoperative AKI and mortality (OR 4.56; 95% CI, 1.40–14.81; *p* = 0.018) (Table 4).

## 4. Discussion

In this study, we investigated the association between intertrochanteric fracture morphology and the incidence of preoperative AKI. Our findings demonstrate that comminuted fracture morphology is an independent risk factor for preoperative AKI. Specifically, patients with comminuted fractures had a 2.44-fold higher risk of developing preoperative AKI compared to those with simple fractures. Moreover, preoperative AKI was significantly associated with prolonged hospital stay and increased in-hospital mortality, underscoring its clinical importance.

Several previous studies have investigated the incidence of AKI following hip fracture surgery, with reported rates ranging from 4.4% to 28.4% depending on study design, and patient population [2,3,4,5,6,7,8,9,10]. For instance, a large population-based cohort study using data from the Danish Hip Fracture Database found that 1717 out of 13,529 patients with hip fracture (12.7%) developed postoperative AKI [2]. Porter et al. [4] reported an AKI incidence of 24.0% (683/2848) during hospitalization following hip fracture. In our study, the incidence of AKI was 14.3% (66/462), which is within the range reported in these previous studies.

However, our study is distinguished from previous studies in several respects. First, we evaluated preoperative AKI, defined as renal dysfunction present at the time of hospital admission, rather than postoperative AKI. This investigation was motivated by our clinical observation that a considerable number of patients with intertrochanteric fractures present to the emergency department with laboratory abnormalities suggestive of AKI, even prior to surgery or medication exposure. Furthermore, by focusing on preoperative AKI, we aimed to investigate the direct contribution of the fracture itself—including trauma-related physiological changes and hidden blood loss—to the development of AKI. Second, we excluded patients with CKD, a major confounding factor, to more accurately assess the development of AKI in patients without baseline renal dysfunction. Third, we focused exclusively on intertrochanteric fractures, unlike most previous studies that included both femoral neck and intertrochanteric fractures. To our knowledge, this is the first study to report the incidence of preoperative AKI in patients with intertrochanteric fractures.

Smith et al. [16] reported that in patients with hip fracture with operative delays greater than 48 h, the mean hemoglobin decrease was 1.49 g/dL for intracapsular fractures and 2.02 g/dL for extracapsular fractures. The authors emphasized the importance of recognizing the risk of preoperative anemia due to hidden blood loss. Stacey et al. [18] also reported that proximal femur fractures are associated with a significant amount of preoperative blood loss. They found that the presence of comorbidities, intertrochanteric fracture, and delayed time to surgery were associated with greater blood loss. Li et al. [17] reported that in patients with intertrochanteric fractures treated with cephalomedullary nail, most of the perioperative hidden blood loss was associated with the initial trauma rather than the surgery. These findings suggest that hidden blood loss in intertrochanteric fracture may contribute to the development of preoperative AKI. Considering that CKD is a well-established risk factor for AKI and that many previous studies included CKD patients, our study showed a relatively high incidence of preoperative AKI (14.3%) despite the exclusion of patients with CKD. This result may be explained by the fact that intertrochanteric fractures—exclusively included in our cohort—are generally associated with greater hidden blood loss compared to femoral neck fractures, which could account for the increased incidence of preoperative AKI observed in our study population.

Additionally, this study is the first to identify the fracture morphology of intertrochanteric fractures as an independent risk factor for preoperative AKI. The proposed pathophysiological mechanism for this association is the increased blood loss typically observed in comminuted fracture. Lin et al. [19] reported that unstable intertrochanteric fractures are associated with a greater drop in hemoglobin during the perioperative period. Excessive blood loss can lead to hypovolemia and hemodynamic instability, both of which are well-established contributors to the development of AKI [1]. In our study, the mean hemoglobin level at admission was significantly lower in the comminuted fracture group, supporting that increased bleeding may contribute to AKI. Although analyzing the change in hemoglobin before and after injury would provide a more accurate estimation of blood loss, this was not feasible due to the lack of pre-injury baseline hemoglobin data in most patients.

CKD, male sex, advanced age, diabetes mellitus, hypertension, cardiovascular disease, the use of angiotensin-converting enzyme (ACE) inhibitors or angiotensin II receptor blockers (ARB), and antithrombotic therapy are reported risk factors for AKI in patients with hip fractures, as identified in prior studies [6,7,10,11,12,13,14,15]. In our study, some of these variables—age, cerebrovascular disease, and antithrombotic agents use—demonstrated borderline associations with preoperative AKI in univariable analysis; however, none remained statistically significant in the multivariable model. This discrepancy may reflect differences in the clinical context and timing of AKI development. Unlike previous studies that primarily focused on postoperative AKI, our analysis targeted AKI present at hospital admission, prior to surgical intervention or exposure to perioperative medications. As such, perioperative factors such as intraoperative blood loss or surgical stress, which contribute to postoperative AKI, were not applicable to our cohort. Additionally, our inclusion criteria—limited to isolated intertrochanteric fractures and excluding patients with chronic kidney disease—may have reduced baseline risk and attenuated the strength of these associations. The use of antithrombotic agents demonstrated a borderline association with preoperative AKI in the final multivariable model (*p* = 0.099). While statistical significance was not reached, this finding suggests a potential link that may become evident with a larger sample size or more detailed categorization. Future studies examining the impact of specific classes of antithrombotic medications on AKI risk may help clarify this association.

Bennet et al. [21] reported that patients with AKI following hip fracture had markedly higher mortality rates, with in-hospital (19% vs. 0%), 30-day (22% vs. 4%), and 120-day mortality (41% vs. 13%). Hong et al. [8] reported that in-hospital mortality was significantly higher in patients with AKI (10.5% vs. 2.3%). Kang et al. [10] reported a one-year mortality rate of 24.0% in the AKI group. Pedersen et al. [2] reported that 30-day (15.9% vs. 5.6%) and one-year mortality (25.0% vs. 18.3%) were significantly higher in patients with AKI. In our study, in-hospital mortality occurred in 12 of 462 patients (2.6%). Patients with preoperative AKI had a significantly higher in-hospital mortality rate (7.6%, 5/66) compared to those without AKI (1.8%, 7/396), demonstrating a similar association between AKI and increased mortality. Our study showed lower absolute mortality rates compared to previous studies, this may be attributed to differences in AKI timing (preoperative vs. postoperative), exclusion of patients with CKD, and improvements in perioperative care. Nevertheless, the relative increase in mortality among patients with AKI remained consistent, emphasizing its prognostic significance even when identified before surgery.

Our findings have important clinical implications. In patients with comminuted intertrochanteric fractures, who are at increased risk of preoperative AKI, early implementation of preventive measures—such as aggressive fluid resuscitation, close renal monitoring, and avoidance of nephrotoxic medications—may reduce the incidence and severity of AKI. Furthermore, given the significant impact of AKI on in-hospital mortality observed in our cohort, early intervention in high-risk patients may contribute to reducing mortality.

This study has several limitations. First, this was a retrospective study conducted at a single hospital, which may limit the generalizability of our findings to broader populations. Second, fracture morphology was categorized using plain radiographs and a relatively simplified classification system, which may introduce misclassification bias. Established systems such as the AO or Evans classification were not used, as our primary focus was on fracture comminution rather than overall fracture stability. Additionally, multiple subgroups of AO classification may limit statistical power and complicate statistical modeling. Third, only a single orthopedic surgeon performed the classification, interobserver reliability could not be assessed and may cause misclassification bias. Fourth, baseline serum creatinine was estimated by assuming an eGFR of 75 mL/min/1.73 m^2^ and applying the MDRD equation. This method may introduce error in elderly patients and potentially overestimate the incidence of AKI, particularly in patients with unrecognized CKD. However, we reviewed all creatinine levels during hospitalization and excluded patients with CKD. Fifth, we did not differentiate between types of AKI (e.g., prerenal vs. intrinsic), which may have provided more insight into the underlying pathophysiology. Fourth, while we evaluated patient comorbidities, we did not account for specific medications (e.g., ACE inhibitors, warfarin) that may influence renal function or hidden blood loss. Finally, long-term clinical outcomes were not evaluated. Due to loss to long-term follow-up in our patients, statistical analysis of 90-day and 1-year mortality was not feasible.

## 5. Conclusions

Comminuted intertrochanteric fracture is independently associated with an increased risk of preoperative AKI in elderly patients. Despite excluding individuals with CKD, the incidence of preoperative AKI remained clinically significant, underscoring the renal vulnerability even in patients without prior renal impairment. Additionally, preoperative AKI was associated with worse clinical outcomes, including longer hospital stays and higher in-hospital mortality. These findings suggest that fracture morphology is a potential risk factor for early renal dysfunction and that careful preoperative evaluation and early supportive management including timely treatment of AKI and close monitoring of renal function may contribute to improving clinical outcomes in this population.

## Figures and Tables

**Table 1:** Comparison of demographic data, clinical outcomes, incidence of preoperative AKI, and preoperative hemoglobin between simple and comminuted fracture groups.

Variable	Simple Fracture (*n* = 205)	Comminuted Fracture (*n* = 257)	*p*-Value
Sex (male/female)	37/168	60/197	0.203 *
Age (years)	82.39 ± 7.75	83.28 ± 7.15	0.202 ^†^
Hypertension	118	168	0.138 *
Diabetes	77	102	0.780 *
History of myocardial infarction	15	25	0.475 *
History of cerebrovascular disease	28	47	0.244 *
Dementia	43	62	0.529 *
Dyslipidemia	82	126	0.081 *
Time to surgery (days)	3.20 ± 3.46	3.52 ± 3.67	0.356 ^†^
Length of hospital stay (days)	15.55 ± 14.18	16.42 ± 11.24	0.474 ^†^
In-hospital mortality	6 (2.9%)	6 (2.3%)	1.000 *
Incidence of preoperative AKI	21 (10.2%)	45 (17.5%)	0.037 *
Preoperative Hemoglobin (g/dL)	11.48 ± 1.86	10.87 ± 1.90	<0.001 ^†^

* chi-square test; ^†^ independent *t*-test.

**Table 2:** Univariable logistic regression analysis of risk factors for preoperative AKI.

Variable	OR	95% CI	*p*-Value
Male sex	0.95	0.41–2.21	0.907
Age	1.04	1.00–1.09	0.077
Comminuted fracture morphology	2.62	1.32–5.23	0.006
Hypertension	1.11	0.35–3.49	0.787
Diabetes	0.95	0.30–3.00	0.887
History of myocardial infarction	0.97	0.31–3.06	0.960
History of cerebrovascular disease	2.16	0.68–6.79	0.095
Dementia	1.12	0.36–3.54	0.749
Dyslipidemia	1.28	0.41–4.03	0.528
Use of antithrombotic agents	1.88	0.60–5.93	0.069
Use of ambulation aid	2.41	1.14–5.08	0.021

**Table 3:** Multivariable logistic regression analysis of risk factors for preoperative AKI.

Variable	OR	95% CI	*p*-Value
Age	1.03	0.97–1.08	0.330
Comminuted fracture morphology	2.44	1.19–5.00	0.015
History of cerebrovascular disease	1.64	0.59–4.59	0.346
Use of antithrombotic agents	1.80	0.89–2.51	0.099
Use of ambulation aid	1.90	0.84–4.29	0.122

**Table 4:** Comparison of clinical outcomes between patients with and without preoperative AKI.

Variable	Preoperative AKI (*n* = 66)	Without Preoperative AKI (*n* = 396)	*p*-Value
Length of hospital stay (days)	20.9 ± 17.0	15.0 ± 11.5	0.001 *
In-hospital mortality	5 (7.6%)	7 (1.8%)	0.018 *

* Fisher’s exact test.

## Data Availability

The data are available from the corresponding author on reasonable request.
